# Tamoxifen and Ovarian Function

**DOI:** 10.1371/journal.pone.0066616

**Published:** 2013-06-28

**Authors:** Martine Berliere, Francois P. Duhoux, Florence Dalenc, Jean-Francois Baurain, Laurence Dellevigne, Christine Galant, Aline Van Maanen, Philippe Piette, Jean-Pascal Machiels

**Affiliations:** 1 Clinique du sein, Centre du Cancer, Cliniques universitaires Saint-Luc, Université catholique de Louvain, Brussels, Belgium; 2 Service d'oncologie médicale, Centre Claudius Regaud, Toulouse, France; 3 Service d'oncologie médicale, Cliniques universitaires Saint-Luc; Institut de Recherche Clinique et Expérimentale (pôle MIRO), Université catholique de Louvain, Brussels, Belgium; 4 Statistical Unit, Centre du Cancer, Cliniques Universitaires St Luc, Université catholique de Louvain, Brussels, Belgium; Davidoff Center, Israel

## Abstract

**Background:**

Some studies suggest that the clinical parameter “amenorrhea” is insufficient to define the menopausal status of women treated with chemotherapy or tamoxifen. In this study, we investigated and compared the ovarian function defined either by clinical or biological parameters in pre-menopausal breast cancer patients treated with tamoxifen administered as adjuvant therapy.

**Materials and Methods:**

Between 1999 and 2003, 138 premenopausal patients consecutively treated for early breast cancer were included. Sixty-eight received tamoxifen in monotherapy as the only adjuvant systemic treatment (Group I) and 70 were treated with tamoxifen after adjuvant chemotherapy (Group II). All patients had a confirmed premenopausal status based on clinical parameters and hormonal values at study entry. They were followed prospectively every 3 months for 3 years: menses data, physical examination and blood tests (LH, FSH, 17-beta-estradiol). Vaginal ultrasonography was carried out every 6 months. After 3 years, prospective evaluation was completed and monitoring of ovarian function was performed as usual in our institution (1x/year). All data were retrospectively evaluated in 2011.

**Results:**

Three patients were excluded from the study in group I and 2 were excluded in group II. Patients were divided into 4 subgroups according to clinical data, i.e. menses patterns. These patterns were assessed by questionnaires. a: Regular menses (>10 cycles/year) b: Oligomenorrhea (5 to 9 cycles/year) c: Severe oligomenorrhea (1 to 4 cycles/year) d: Complete amenorrhea Estrogen levels did not appear to have any impact on disease-free survival rates after 3 or 8 years. FSH values were also documented and analyzed. They exhibited the same profile as estradiol values.

**Conclusions:**

Amenorrhea is an insufficient parameter to define menopausal status in patients receiving tamoxifen. Low estradiol levels must be coupled with other biological parameters to characterize endocrine status. These data are very important for the choice of endocrine therapy.

## Introduction

Adjuvant endocrine therapy reduces tumor recurrence and mortality in pre- and postmenopausal women with hormone receptor-positive early breast cancer [1], [2], [3]. Before the aromatase inhibitor era, tamoxifen was the accepted adjuvant endocrine therapy for both pre- and postmenopausal women with hormone receptor-positive breast cancers. Today, tamoxifen remains the standard of care for premenopausal women. However, in the first Early Breast Cancer Trialists' Collaborative Group (EBCTCG) meta-analysis [4], tamoxifen appeared to provide little benefit for premenopausal women. For this reason, many trials on adjuvant therapy in premenopausal women subsequently focused on ovarian ablation and chemotherapy [4], [5], [6]. Later EBCTCG [6] updates showed substantial benefits of tamoxifen in women under 50 years of age with hormone receptor-positive disease. Results revealed a 45% reduction in the risk of recurrence and a 32% decrease in the risk of death, irrespective of chemotherapy. Trials on tamoxifen associated with ovarian function suppression have also been conducted but the results are still unknown and data from some trials will probably be published in the course of 2013. Furthermore, it has not yet been established whether the use of an aromatase inhibitor with ovarian function suppression is superior to tamoxifen alone or tamoxifen plus ovarian function suppression. The benefits and risks of these associations will be better defined when the results from the SOFT and TEXT trials will be available.

In this study, we investigated and compared the ovarian function defined either by clinical or biological parameters [7], [8], [9] in pre-menopausal breast cancer patients treated with tamoxifen administered as adjuvant therapy.

## Materials and Methods

### Ethics statement

Our study is an observational study for which we obtained the agreement of our ethics committee (Commission d'éthique biomédicale Hospitalo-Facultaire de l'Université catholique de Louvain, date of agreement 4th April 2011). Written consent given by the patients was not needed because our observational study included patients between 1999 and 2003 before the implementation by the Belgian parliament on 7th May 2004 of the European directive 2001/20/EC (http://eur-lex.europa.eu/LexUriServ.do?uri=CELEX:32001L0020:en:NOT). The data were analyzed anonymously and patients could not be identified from the unique study number that they were assigned. No additional laboratory tests were performed beyond those recommended by local guidelines.

### Inclusion criteria

Eligible patients were required to have histologically or cytologically proven early breast cancer treated with curative-intent surgery, estrogen (ER) and/or progesterone (PR) receptors detected on the tumor by immunohistochemistry, no distant metastases, and a confirmed premenopausal status defined as estradiol >40 pg/ml, LH <18.7 mUI/ml, and FSH <45.7 mUI/mL. (values defined by our laboratory) before the initiation of adjuvant chemotherapy or tamoxifen. In group II, the entry point was determined by recovery of premenopausal hormone values (some patients had already received tamoxifen for 2 or 3 months).

Menopausal values were defined by high FSH values >45,7 mUI/ml, low levels of estrogens and clinical amenorrhea >one year.

### Study design

This was a retrospective monocentric study performed at Cliniques universitaires Saint-Luc (Brussels). Patients were followed prospectively every 3 months for 3 years: menses data, physical examination and blood tests (LH, FSH, 17-beta-estradiol). To observe cyclic changes of hormonal values, samples were taken on different days, at different points of the cycle.Vaginal ultrasonography was carried out every 6 months. After 3 years, prospective evaluation was completed and monitoring of ovarian function was performed as usual in our institution (1x/year). All data were prospectively collected.

### Statistical analysis

Statistical analysis was performed using the Welch two sample t-test (comparison of hormone values) between subgroups. p-values <0.05 were considered as statistically significant. Survival and disease-free survival were calculated from the date of surgery using the Kaplan-Meier method. A Cox proportional hazard ratio test was also used to evaluate the interaction of estrogen variations and disease-free survival or the absence of interaction.

### Endpoints

The main aim of the study was the comparison between ovarian function defined by clinical *vs.* biological parameters in pre-menopausal breast cancer patients treated with tamoxifen administered as adjuvant therapy.

A secondary objective was the evaluation of the impact of high *vs.* low estrogen levels on disease-free survival after 3, 5 or 8 years in premenopausal breast cancer patients receiving tamoxifen.

To define menopausal status and premenopausal status, we used the values of our laboratory that correspond to hormonal values usually used in Europe. The values of the NCCN (National Comprehensive Cancer Network) were for menopausal status FSH values >40 UI/l, low estrogen levels and absence of menses for more than one year.

## Results

### Patients' characteristics

Patients' and tumor characteristics are summarized in table 1. Between 1999 and 2003, 138 premenopausal patients treated for early breast cancer were included in the study. Sixty-eight patients received tamoxifen 20 mg as the only systemic adjuvant treatment (Group I) and 70 were treated with tamoxifen 20 mg after adjuvant chemotherapy (6 cycles of FEC 100 on day 1 every 3 weeks [5-fluorouracil 500 mg/m2, epirubicin 100 mg/m2 and cyclophosphamide 500 mg/m2] or 4 cycles of EC on day 1 every 3 weeks [epiadriamycine 75 mg/m2 and cyclophosphamide 500 mg/m2]) (Group II). Radiation therapy was systematically administered after conservative surgery and was only administered after mastectomy if lymph nodes were invaded.

**Table 1:** Patients' and tumor characteristics.

	*Group I*	*Group II*
N° of patients	68 (3 excluded) –65	70 (2 excluded) –68
T stage		
T1a	4	0
T1b	53	2
T1c	8	40
T2	0	26
Grade		
Grade I	21	10
Grade II	41	32
Grade III	3 (all T1a)	26
Hormone receptors		
ER and PR +	62	64
ER + and PR –	3	3
ER – and PR +	0	1
Margins	Free	Free
Type of surgery		
Mastectomy	1	20
Lumpectomy	64	48
Age	36 to 48 (mean 44.5)	32 to 47.5 (mean 42)
Lymph nodes		
N0	65/65	34 (stage I)
N1		34 (stage II)
1 to 3 nodes		32
>3 nodes		2
Histology		
Infiltrating lobular	7	8
Infiltrating ductal	58	60
Number of pregnancies		
0	7	6
1	15	13
2	25	30
3	13	11
>3	5	8
Chemotherapy	0/65	68/68
4 EC		34
6 FEC		34
*		EC FEC
Re	6	
–o		
Radiotherapy	64/65	65/68

ER: estrogen, PR: progesterone, * data after adjuvant chemotherapy for group II.

Median age was 44.5 years (range: 36–48) in group I and 42 years (range: 35–47.5) in group II. In group I, all patients were American Joint Committee on Cancer, 2009 (AJCC) stage I, while in group II, 34 were AJCC stage I and 36 AJCC stage II. Three patients were excluded from the study in group I (2 underwent hysterectomy for benign myomas and 1 received an LHRH agonist for recurrent ovarian cysts) and 2 were excluded in group II (1 received an LHRH agonist for ovarian cysts and 1 showed metastatic evolution after 10 months).

### Clinical and biological menopausal status

Menopausal status is a major consideration in adjuvant [7] breast cancer therapy. Unfortunately, there is no universal definition of menopausal status.

According to the clinical data about their bleeding patterns (table 2), patients were first classified in 2 groups: patients with regular menses and patients with irregular menses. In an attempt to classify more precisely bleeding irregularities, patients were subsequently divided into 4 subgroups: (a) regular menses (≥10 cycles/year), (b) oligomenorrhea (5 to 9 cycles/year), (c) severe oligomenorrhea (1 to 4 cycles/year), and (d) complete amenorrhea. Data about the bleeding patterns were available for all patients before the start of any breast cancer treatment. For patients in group II, data were available at the end of chemotherapy and for all patients, data were available during tamoxifen treatment. Before beginning any treatment, 60/65 patients (92.3%) in group I had regular menses and 5 patients (7.7%) exhibited oligomenorrhea. In group II, 65 patients had regular menses (95.5%) and 3 (4.5%) patients mentioned oligomenorrhea. For patients receiving chemotherapy, data indicated that in the EC arm, 31/34 patients (91.1%) recovered regular menses before receiving tamoxifen and 3 patients (8.9%) had oligomenorrhea. In the FEC arm (34 patients), 5 patients (14.7%) recovered regular menses, 5 patients had oligomenorrhea (14.7%) and 24 patients (70.6%) were still amenorrheic at study entry. While on tamoxifen therapy, the number of patients in each subgroup (a, b, c and d) was 3 (4%), 19 (29%), 38 (55%) and 5 (8%) in group I (65 patients) and 2 (3%), 21 (30%), 38 (55%) and 7 (10%) in group II (68 patients), respectively.

**Table 2:** Menses patterns.

	Group I	Group II	
N°of patients	65	68	
Pretreatment data			
- Regular menses	60	65	
- Oligomenorreha	5	3	
Postchemotherapy data		EC arm (n = 34)	FEC arm (n = 34)
- Regular menses		31	5
- Oligomenorrhea		3	5
- Amenorrhea			24
While on tamoxifen treatment			
- Regular menses	3	2	
- Oligomenorrhea	19	21	
- Severe oligomenorrhea	38	38	
- Amenorrhea	5	7	

**Figure 1:**
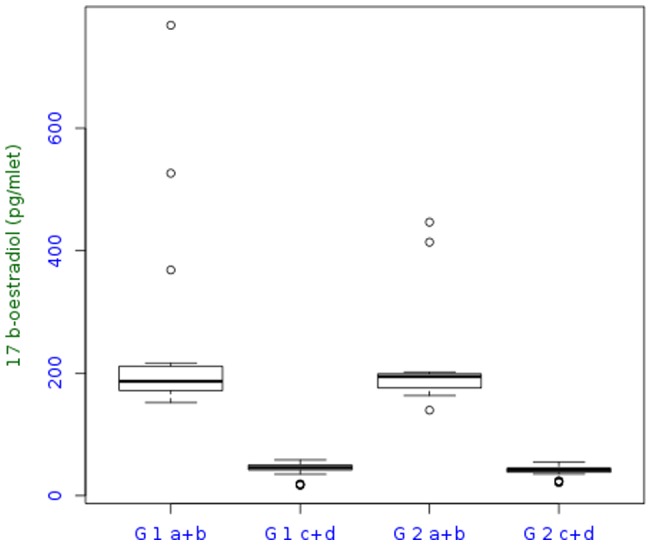
Median 17-beta-estradiol level.

**Figure 2:**
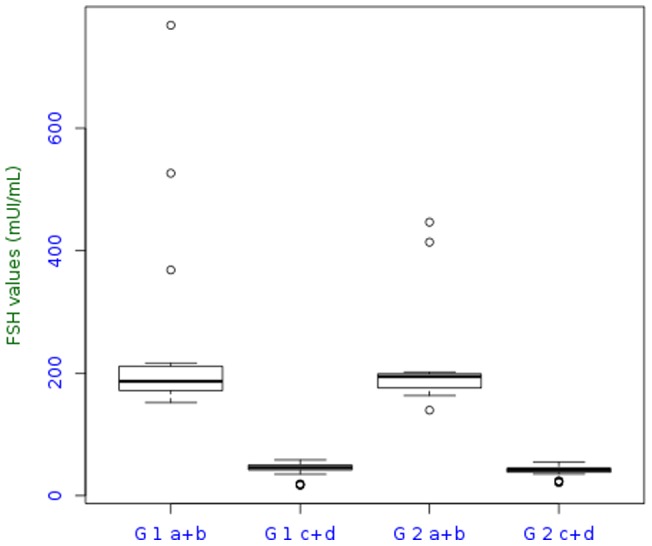
Median FSH level.

Hormonal values are summarized in Table 3 and shown in Fig 1 and 2. The most interesting parameter seemed to be the estradiol levels (Table 3).

**Table 3:** Hormonal Values

Median and range estrogen values		
	Group I	*Group II*
Subgroup a	315 (48–1150) pg/ml	295 (40–995) pg/ml
Subgroup b	198 (49–380) pg/ml	190 (58–370) pg/ml
Subgroup c	58 (12–90) pg/ml	60 (10–84) pg/ml
Subgroup d	24 (10–30) pg/ml	21 (10–32) pg/ml
**Median and range FSH values**		
Subgroup a	20 (6–25) mIU/ml	15.8 (4.3–20.5) mIU/ml
Subgroup b	17 (3–24.1) mIU/ml	18.8 (4–254) mIU/ml
Subgroup c	2.5 (1–4) mIU/ml	2.8 (0.5–4.8) mIU/ml
Subgroup d	1.1 (0.5–20.7) mIU/ml	1 (0.6–1.3) mIU/ml

For patients in subgroup a, tamoxifen mimicked ovulatory cycles such as those observed in patients receiving clomiphene citrate (regular menses with high levels of estradiol). In contrast, for patients in subgroup b, hormonal variations corresponded to a shift between normal spontaneous ovulatory and anovulatory cycles.

For patients in subgroups c and d, tamoxifen induced low estradiol, LH and FSH, responsible for severe oligomenorrhea or complete amenorrhea.

All patients, after cessation of tamoxifen, recovered menses and have samples of FSH taken on day 3, showing low FSH values (confirmation of premenopausal status).

For all patients of stage I (group I n = 65 patients and group II n = 34 patients), no impact of estrogen levels on DFS was observed: p = 0.877. For patients of group II, (34 patients stage I and 34 patients stage II), the same analysis was performed and the results confirmed that the variations of estrogen levels induced by tamoxifen had no statistically significant impact on DFS: patients of stage I p = 0.682 and patients of stage II p = 0.271.

### Disease-free survival and overall survival

**Figure 3:**
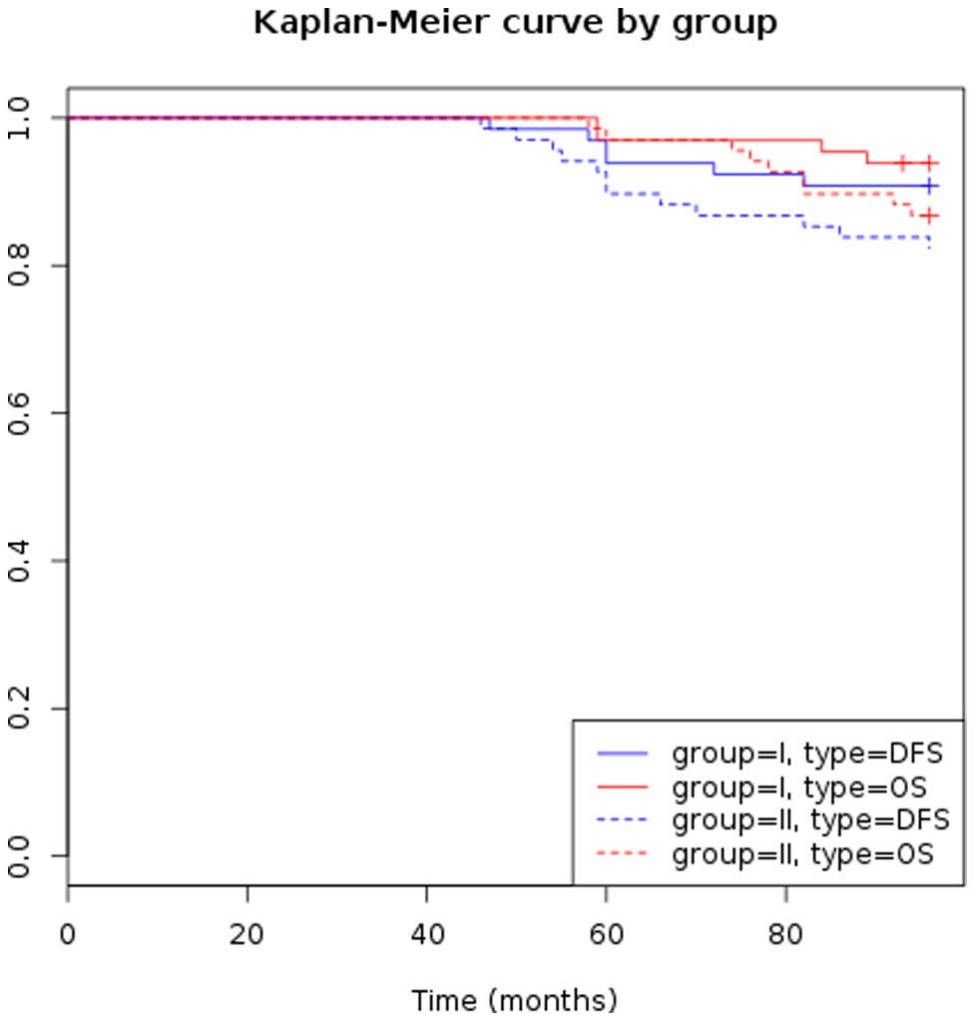
Disease-free survival and overall survival in group I and group II.

**Figure 4:**
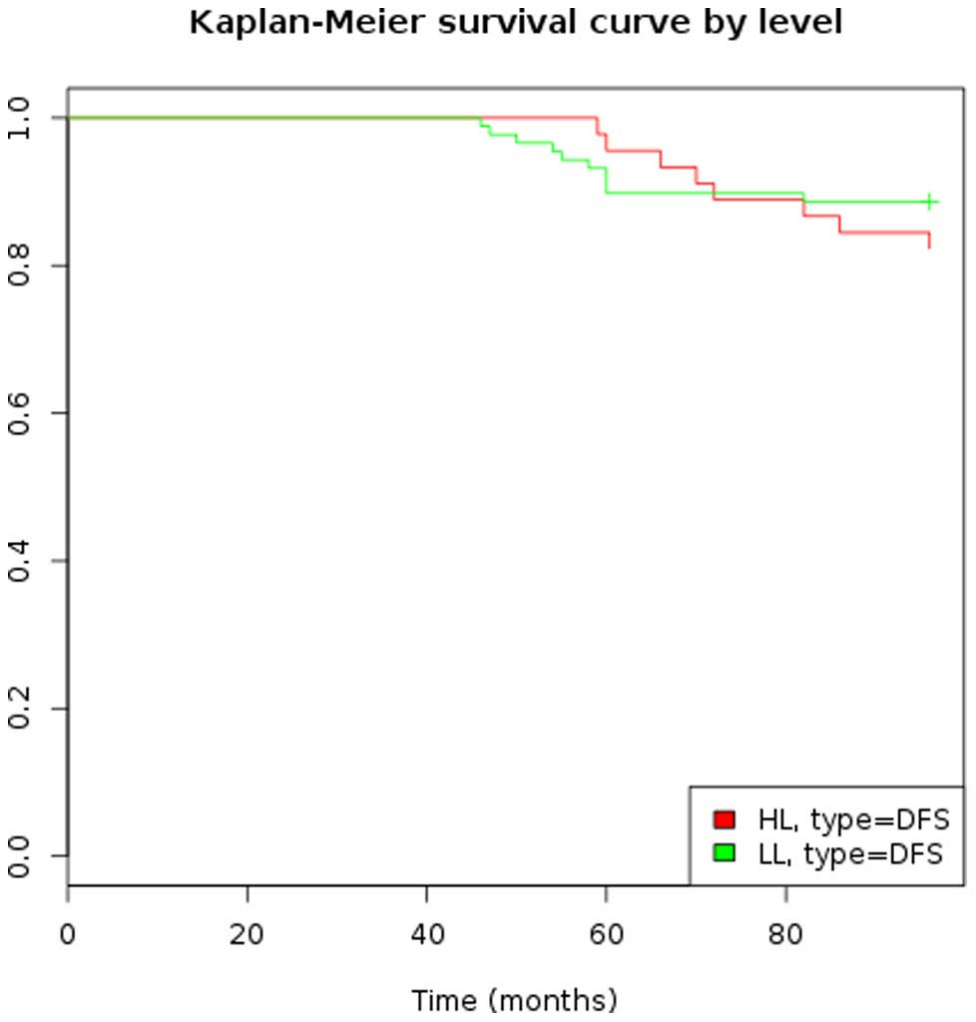
Disease-free survival according to 17-beta-estradiol level.

Disease-free survival and overall survival of groups I and II are shown in Figure 3 and were in the expected ranges. No statistical difference concerning disease-free survival was observed between patients with high estrogen levels (subgroups a and b) and those with low estrogen levels (subgroups c and d) (figure 4).

## Discussion

In this study, all patients presenting with breast cancer were premenopausal. Their premenopausal status was confirmed by hormone values associated with clinical signs. For patients not receiving chemotherapy, determination of premenopausal status before tamoxifen is straightforward, but for those receiving chemotherapy, it is more problematic. It is known that adjuvant chemotherapy can induce chemotherapy-related amenorrhea, which temporarily suppresses menses [2], [10], [11], [12], [13], [14]. It is therefore very important to obtain hormonal values associated with information about the presence of menses. Adjuvant chemotherapy is also thought to indirectly induce a temporary or complete ovarian suppression. Thus, the presence or absence of menses is insufficient to demonstrate menopausal or premenopausal status, and biological data are required. In case of biologically confirmed premenopausal status, contraception is required while on tamoxifen treatment.

In our study, it was interesting to note that patients receiving tamoxifen both alone and after chemotherapy showed similar menses patterns with a similar distribution of patients between regular and irregular menses. In group I, 22/65 patients had regular menses or light oligomenorrhea, while 23/68 patients exhibited the same pattern of menses in group II. As for severe oligomenorrhea and complete amenorrhea, the numbers were respectively 43 patients in group I and 45 patients in group II. Therefore, chemotherapy did not appear to influence ovarian response to tamoxifen, which exerted its own effect, independently of cytotoxic therapy

Tamoxifen may increase plasma estrogen concentrations by interfering with normal negative pituitary feed-back mechanisms [14], [15]. The impact of tamoxifen on ovarian function is not well understood, especially since the number of premenopausal patients not receiving adjuvant chemotherapy in the literature is very small. In a meta-analysis by the EBCTCG published in 1991 [4], only 107 premenopausal patients were included in evaluated studies. The 2005 update of the EBCTCG meta-analysis of tamoxifen trials yielded consolidated data on hormonal treatment of premenopausal patients. Data on the interaction of tamoxifen with ovarian function are nevertheless seriously lacking in the literature [16], [17]. In an old study by Manni [15] on metastatic breast cancer patients, the effect of tamoxifen on menstrual cycles ranged from no effect at all to complete cessation of menses. However, in this small study (only 11 patients), the authors observed that amenorrhea was more frequent in patients receiving high doses of tamoxifen (up to 120 mg), so they proposed escalating doses of anti-estrogens to avoid high levels of estrogens. We know that estrogen levels can increase 3–10 fold with tamoxifen use depending [16], [17] on the time in the cycle it is given. It also seems to be variable from patient to patient. The rise in estrogen levels could theoretically interfere with the anti-tumoral effects of tamoxifen because the latter competitively inhibits estrogen binding to its receptor. There is however no evidence in the literature that this occurs or has any impact on the benefits of tamoxifen therapy in premenopausal women. The results of meta-analyses published after 1995 [6] are similar in both premenopausal and postmenopausal patients. The decreased risk of recurrence in patients under +/−50 years of age is 27+/−7%, while the reduction in the odds of death is 17+/−10%. For patients aged 50 years or more, the reduction in the annual odds of recurrence is 30+/−2% and in the annual odds of death 19+/−3%. Results of the different meta-analyses of the EBCTCG have yielded consolidated data on hormonal treatment of premenopausal patients, but data on the interaction of tamoxifen with ovarian function are rarely published in the literature [16], [17], [18]. It is known that tamoxifen may increase plasma 17-beta-estradiol concentrations by interfering with normal negative pituitary feed-back mechanisms, with a resulting increase in FSH-driven ovarian steroidogenesis [19].

However, there may be additional mechanisms involved, including direct interaction of tamoxifen with granulosa cells, increased FSH concentrations and thus estrogen production, as well as modified LH receptor expression (studies published by Murray and Rafdim) [1], [17]. Amenorrhea is therefore an insufficient parameter to define menopausal status in patients receiving tamoxifen, and it becomes even more problematic in patients who have undergone chemotherapy, because chemotherapy-related amenorrhea is very frequent [14]. Patients receiving 4 cycles of EC treatment recovered menses in almost all cases before beginning endocrine treatment, but those undergoing 6 cycles of FEC chemotherapy recovered menses only later, which is why it is necessary to use biological parameters. The most common biomarkers used in this study were FSH and estradiol. We did not test anti-Müllerian hormone (AMH) or inhibin B, as data published in the literature are still controversial: some studies show interesting results with AMH and inhibin, but others reveal inconsistencies [20], [21]. Another hypothesis to explain the pattern of menses observed with tamoxifen treatment could be the potential role of pharmacogenomics, with an impact of CYP2D6 [22], [23], [24], [25], [26]. An explanation for the different patterns of ovarian function observed while on tamoxifen treatment will perhaps be found in the metabolization of tamoxifen. Indeed, tamoxifen is a prodrug that is metabolized to active metabolites: N-desmethyl-tamoxifen, 4-hydroxy-N-desmethyl-tamoxifen (endoxifen) and 4-hydroxytamoxifen. These metabolites have up to 33 times more affinity for the estrogen receptor than tamoxifen. Both endoxifen and 4-hydroxytamoxifen have similar potency, in vitro and in vivo, to both the ERa and ERb receptors. They have a similar dose-response relationship on several ER-positive breast cancer cell lines. Endoxifen is also a potent antagonist of the progesterone receptor gene, with similar potency than 4-hydroxytamoxifen. Among the active metabolites, endoxifen is probably the most clinically relevant in terms of hormone receptor blockade, since its plasma concentration is 5–10 times higher than that of 4-hydroxytamoxifen. We did not evaluate endoxifen or 4-hydroxytamoxifen in our study, but this probably constitutes a very interesting approach that needs to be investigated in prospective studies.

### Conclusion

Amenorrhea while on tamoxifen does not, in itself, confirm definitive menopause. Ovarian response to tamoxifen exhibits different patterns, not found to be influenced by previous administration of chemotherapy in our study. Tamoxifen metabolization looks to be an interesting tool that needs to be further investigated in prospective studies in an attempt to explain ovarian response to tamoxifen.
