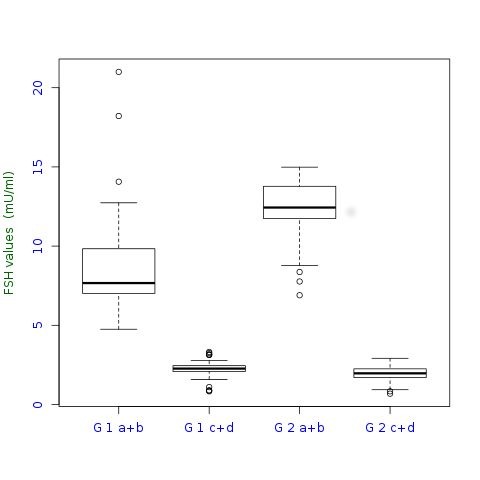# Correction: Tamoxifen and Ovarian Function

**DOI:** 10.1371/annotation/b6747d3d-f41b-4421-abd8-f32def535dde

**Published:** 2013-10-10

**Authors:** Martine Berliere, Francois P. Duhoux, Florence Dalenc, Jean-Francois Baurain, Laurence Dellevigne, Christine Galant, Aline Van Maanen, Philippe Piette, Jean-Pascal Machiels

Figure 2 is a duplicate of Figure 1. Please see the correct Figure 2 here: 

**Figure:**